# The Lived Experiences of COVID-19 Patients in South Korea: A Qualitative Study

**DOI:** 10.3390/ijerph18147419

**Published:** 2021-07-12

**Authors:** Haeng-Mi Son, Won-Hee Choi, Young-Hui Hwang, Hye-Ryun Yang

**Affiliations:** 1 Department of Nursing, University of Ulsan, Ulsan 44610, Korea; sonhm@ulsan.ac.kr (H.-M.S.); hyh77@ulsan.ac.kr (Y.-H.H.); 2 Department of Nursing, University of Kyungsung, Busan 48434, Korea; whchoi@ks.ac.kr; 3 Gyeongnam Center for Infectious Disease Control and Prevention, Changwon 51154, Korea

**Keywords:** COVID-19, qualitative research, quarantine, Korea, stigma

## Abstract

The purpose of this qualitative study is to provide an in-depth understanding and description of the disease experiences of COVID-19 patients. The participants were 16 patients discharged from hospitals after receiving treatment for COVID-19 in isolation. Data collection was conducted through individual in-depth interviews until data saturation, and the interviews were recorded and transcribed verbatim. Data were analyzed using Colaizzi’s phenomenological method. The participants were quarantined after their COVID-19 diagnosis was confirmed, and they experienced desperate and uncertain times during treatment. The participants expressed shock and dissatisfaction due to an excessive invasion of privacy during the quarantine process and in the quarantine system. As confirmed COVID-19 cases, the participants experienced social stigma and feelings of guilt, negative attitudes from others and society, and negative influences from social networking services and the media. The participants also experienced mental and physical difficulties due to COVID-19 symptoms. However, they rediscovered meaningful relationships through the support of their family and friends in the midst of adversity. It is necessary to provide an integrated psychosocial rehabilitation program to reduce social stigma and improve the resilience of COVID-19 patients.

## 1. Introduction

With the number of coronavirus disease 2019 (COVID-19) diagnoses and deaths increasing rapidly worldwide [1,2], COVID-19, which was declared a pandemic in March 2020 by the World Health Organization, has brought a tremendous amount of shock and changes to all aspects of societies worldwide—spanning from politics to economics—so much so that the time following the beginning of the COVID-19 pandemic is now called the “post-COVID-19 era” [3,4]. Despite aggressive measures implemented in countries worldwide to prevent the massive spread of COVID-19, the virus is anticipated to be difficult to eradicate until the development of effective vaccines and therapeutic agents [4,5].

In response to the increasing number of COVID-19 patients globally, many studies have examined their physical symptoms and changes in mental conditions [6,7,8,9]. COVID-19 patients may experience physical symptoms such as fever, dyspnea, cough, and adverse drug reactions [6] as well as mental symptoms, such as fear of contracting the novel virus, loneliness, anger related to receiving treatment in isolation, and post-traumatic stress [6,7]. It has been reported that even if COVID-19 patients are asymptomatic or have mild symptoms, they might experience fear of death or mental distress due to isolation [8]. Furthermore, COVID-19 patients who have spread the infection to others may even experience guilt [9]. During the early days of the COVID-19 pandemic, various channels, such as mobile messengers and YouTube, frequently described the virus as being highly dangerous, with a poor prognosis [7], and public media coverage of COVID-19 intensified the public’s fear and anxiety [10].

South Korea was able to halt the massive spread of COVID-19 nationwide through conducting intensive contact tracing, implementing timely diagnosis and treatment, and promoting national physical distancing campaigns [11]. However, although the so-called “K-Disinfection model” gained worldwide attention, it is speculated that the excessive collection and disclosure of personal information involved in these aggressive disinfection measures have had a detrimental impact on COVID-19 patients [3]. As South Korea is a high-context society that shares extensive information and emphasis on relationships [12], COVID-19 can entail significant physical, psychological, and social meaning beyond simply contracting an illness. However, most previous studies have only reported these patients’ physical symptoms and changes in mental conditions [6,7] without sufficient depth to provide a comprehensive understanding of the subjective disease experiences of COVID-19 patients.

As phenomenological qualitative studies are conducive to examining meaning in individually perceived and interpreted experiences [13], such a study design is appropriate for gaining an in-depth understanding of the meaning in the disease experiences of COVID-19 patients in South Korea. Thus, this study aimed to conduct and examine phenomenological qualitative interviews to describe the disease experiences of COVID-19 patients in depth and to understand the meaning and essential structure of their physical, psychological, and social stress. We hope this study’s findings will contribute to the development of an intervention program that promotes and supports comprehensive recovery (i.e., physical, psychological, and social) among COVID-19 patients.

## 2. Materials and Methods

### 2.1. Study Design

This study used a qualitative phenomenological design. A phenomenological study is a form of qualitative research, with its epistemological grounds based on phenomenology, and it allows for the exploration of individuals’ lived experiences through researchers’ phenomenological reduction examine the meaning and essential structure of these experiences. Thus, we deemed this methodology to be appropriate to explore the following research question: “What are the meanings and essence of the disease experiences of COVID-19 patients?”

### 2.2. Participant Selection

We used purposive sampling to recruit 16 adults with a prior history of COVID-19-related hospitalization who were able to read and communicate in Korean. The research team obtained a list of COVID-19 patients who expressed willingness to participate in the study from a public official in charge at the public health center through a formal procedure with a local health and administration agency. We contacted the participants via telephone and reconfirmed their consent before enrolling them in the study. The participants were in their 20s to 70s, with nine men and seven women enrolled in the study. At the time of hospitalization, 14 were symptomatic, while 2 were asymptomatic, and the route of infection varied widely, including religious facilities, work, and relatives. The mean length of hospital stay was 34 days, and 13 had sequelae, while 3 did not (Table 1).

### 2.3. Researcher Characteristics and Reflexivity

The researchers are experts in qualitative research or infection control who share a consensus on the need to explore the phenomenon of interest. The researchers have rich knowledge and understanding of the collection and analysis of qualitative data based on their experiences conducting multiple qualitative studies. To avoid their specializations from becoming a bias during the study, the researchers interacted as active listeners while collecting the data and shared and discussed their notes with each other throughout the study procedure.

### 2.4. Data Collection

Individual in-depth interviews were conducted by researchers in a conference room at the public health center or hospital at the participant’s convenience and lasted approximately 1–2 h. All interviews were audio-recorded. The key interview questions were as follows: “How did you feel when you received the COVID-19 screening test and were confirmed to have COVID-19?”, “How was your experience with the disinfection procedures and hospitalization in an isolation ward after being diagnosed with COVID-19?”, and “How did COVID-19 affect you psychosocially?” The interviews began with small talk to build a rapport with each participant. Data were collected until saturation was reached, and the interview recordings were transcribed. We obtained written consent from the participants and collected general information before beginning the interviews. Data were collected between September and November 2020.

### 2.5. Data Analysis

Data were analyzed using the phenomenological method proposed by Colaizzi [14]. We read the data from each interview and coded significant statements. The principal researcher collected and organized the coded statements. Then, we derived and refined significant statements, themes, and theme clusters through several rounds of discussion. The detailed data analysis process is as follows. First, we read the interview transcripts several times to familiarize ourselves with the data and extracted significant statements directly related to the study phenomenon. Then, we formulated broader meanings from the significant statements while bracketing our presuppositions as much as possible. Similar meanings were then clustered into themes, and similar themes were integrated into theme clusters. Next, we wrote a comprehensive description of the phenomenon, incorporating all revealed themes, and condensed the exhaustive descriptions into dense statements to capture the essential structure of the phenomenon. We reviewed all steps of the analysis procedure to confirm that they sufficiently captured the essential structure of the participants’ experiences.

### 2.6. Ethical Considerations

This study was approved by the institutional review board at the first researcher’s affiliated university (1040968-A-2020-014). We provided an information sheet explaining the study to the participants and obtained their written consent. Specifically, we informed the participants that the interviews would be recorded, the collected data would be coded with participant IDs to protect personal information and stored in a locked file on a computer, and they had the freedom to withdraw from the study at any time.

### 2.7. Trustworthiness

To ensure the trustworthiness of the research, we applied the criteria for credibility, fittingness, auditability, and confirmability [15]. To establish credibility, participants with abundant COVID-19 experience were purposively sampled, and data were collected until saturation. Furthermore, the participants’ statements were audio-recorded and transcribed verbatim, and the transcripts were reviewed against the recordings to ensure the accuracy of the text. After we described the essence of COVID-19 experiences based on the participants’ interviews, the participants were shown the results and asked to verify whether the descriptions accurately captured their experiences. Results in Korean were translated by a Korean–English bilingual translator and checked by the researchers to minimize any potential meaning distortions in the translation process. To establish fittingness, participants were selected in consideration of various demographic- and COVID-19-related factors to expand the context of the COVID-19 experience. Auditability and confirmability were established by strictly complying with each step of Colaizzi’s phenomenological method [14], and the researchers collected and circularly analyzed data with phenomenological reduction and strived to maintain neutrality toward the study phenomenon throughout the process. The final theme clusters were determined based on several rounds of discussions among the researchers. Data analysis was performed manually using a word processor. The logical flow and comprehensiveness of the study results were reviewed by a qualitative research expert who was external to the research team. The entire process of the study was conducted according to COREQ [16].

### 2.8. Limitations of the Findings

This study enrolled patients from one region in South Korea who had been diagnosed with COVID-19 early in the pandemic; thus, the experiences of our participants may differ from those of patients who contracted the virus after COVID-19 disinfection measures were relatively settled.

## 3. Results

In this study, 75 significant statements, 18 themes, and 5 theme clusters emerged (Table 2).

### 3.1. Theme Cluster 1: Desperate and Uncertain Times during COVID-19 Diagnosis and Treatment

#### 3.1.1. Feeling Anxious about COVID-19 Diagnosis

Most participants took the COVID-19 screening test because they had developed a high fever. Although they took the screening test after careful contact tracing and suspicion of COVID-19, their true intention was to rule out COVID-19, as opposed to being diagnosed with it, and to stop worrying about it. While waiting for the test results, the participants felt anxious and nervous about being diagnosed with COVID-19. Even while hoping that they had not contracted the infection, they intuitively knew that they could have. Receiving the confirmation of COVID-19 infection was an unexpected disaster.

*“I was worried because I didn’t know whether I have confirmed COVID-19 or not. I think I’m not (COVID-19 confirmed), but I don’t know what the results will be.”*

(Participant 10)

#### 3.1.2. Terrifying Life in Quarantine Room with Everything Blocked

While living on the isolation ward, the participants had to sever all outside communication and were deprived of their freedom. The participants described the environment as being similar to confinement, jail, imprisonment, restriction, and animal pens. They were downhearted regarding the physical weakness brought on by the infection and felt lonely and scared alone on the ward. The negative-pressure wards were dry and cold, and the participants missed the fresh air and landscape of the outside world. Most significantly, all visitors were prohibited, and their cellphones were the participants’ only means of communication. Some participants also discussed the positive aspects of isolation, stating that it offered an opportunity to reflect on their lives. Most participants were admitted in the winter and discharged in the spring, and as they recalled the long hospital stay that spanned several months, they felt relieved to have survived and grateful for regaining their freedom.

*“I just lost my temper. I was like, this isn’t even a disease, and why are you keeping me here? Give me proof I don’t have the disease—give it to me.”*

(Participant 4)

#### 3.1.3. Conquered by Helplessness

Aside from when the participants were treated for COVID-19 symptoms, most of the time on the ward was meaningless repetition while waiting for negative COVID-19 results and discharge, which seemed perpetual and boring. The participants felt helpless, resulting in even more stress.

Some participants engaged in small activities such as laundry, exercise, reading, drawing, writing, listening to music, religious activities, and games to relieve the boredom and helplessness they felt in the ward.

*“I asked my dad to bring me my ukulele. So, he brought it, and I played it. Playing an instrument seemed to relieve my stress. Just the fact that I could do something in there relieved my stress and relaxed my body.”*

(Participant 3)

#### 3.1.4. Appreciation for Medical Personnel

The participants were grateful for the health care providers who took care of them while risking infection, and they depended on health care providers who consoled them with kind words. In particular, they felt sorry for the nurses as they watched them work tirelessly in heavy anti-contamination clothing and gear. Some participants tried to lessen nurses’ burden by doing some chores on their own, such as dumping their trash. In contrast, some participants complained about ineffective communication with health care providers and inappropriate treatment due to these providers visiting infrequently.

*“I think the nurses probably suffered the most. They would sweat like crazy, and you could see that their goggles were foggy from the sweat. I felt so sorry. I mean, wouldn’t they be horrified (of getting COVID-19), too? It must’ve been so terrifying, but when they come in all sweaty, they would try to only say good things and not say bad things. I think that mattered a lot.”*

(Participant 10)

### 3.2. Theme Cluster 2: Shock and Complaints Related to the Disinfection Process

#### 3.2.1. Being Completely Stripped of Privacy during the Disinfection Process

The participants were infuriated at the disinfection measures that utterly disregarded the patients themselves and their families. Once COVID-19 was confirmed, admission to a health care facility and disinfection of the patient’s residence occurred immediately, night or day. The sight of an ambulance with blaring sirens and disinfection personnel in their protective gear announced to the entire neighborhood that the participants had contracted COVID-19.

*“So, I went to the hospital, and people who weren’t transferred don’t know how that felt. It even rained when I was taken. They disinfected the entire house, and I went outside, and I thought about what this would look like to people in my neighborhood. It’s not like I was sick with something else. I caught an infectious disease.”*

(Participant 13)

In particular, the disclosure of personal information and thorough contact tracing reported in the press or on social media was absolutely terrifying and a downright violation of privacy. The repercussions of disclosing information about confirmed patients were serious in the early days of the COVID-19 outbreak. One participant said that it was very embarrassing, and he likened himself to a biblical story of a woman who had committed adultery. Further, the participants were offended by the oppressive attitudes of the epidemiological investigators, who displayed mistrust and interrogated them despite the fact that they were fully cooperative with the investigation. They felt like they were being treated as convicted criminals by the police. Having to talk about places they had gone when they could not remember them well was the most difficult. As they were stripped of their personal privacy during the disinfection procedure, the participants asserted that even confirmed patients need to be protected as any other citizen would be.

*“My name, someone told me that my name was out everywhere. My work and department, my apartment, how many kids I have, even my son and daughter. They saw it in the news. I felt like I was being stripped naked. I felt that they were just throwing me out of this land.”*

(Participant 12)

#### 3.2.2. Unorganized Disinfection System

Disinfection measures in the early days of the COVID-19 outbreak were not firmly established, and even the relevant public officials were confused about the system. The participants were frustrated by the impractical disinfection guidelines and manuals enforced by infection management personnel with no flexibility. Due to the lack of a single epidemiological investigation facility and system, the participants had to respond to several epidemiological investigators repeatedly in each relevant facility, amid confusion and chaos, which aggravated their stress.

*“I got three calls, from epidemiological investigators, a public health center, and the Korea Disease Control and Prevention Agency (KDCA). It was lunch time, but I couldn’t eat. I couldn’t even eat dinner. I was taking these calls, and I talked to the point that my mouth tasted bitter. The same thing, repeatedly.”*

(Participant 10)

#### 3.2.3. Need for Government Policies

The participants stated that detailed disinfection guidelines needed to be applied to specific situations. They suggested that asymptomatic and symptomatic patients should be separated, and quarantine requirements should vary according to the patient’s condition. They also mentioned the problems of epidemiological investigations that do not take the incubation period into consideration. In particular, they pinpointed the severe financial repercussions suffered by individuals and businesses due to the strictly uniform disinfection guidelines applied during the early days of the COVID-19 outbreak and stated that the criteria for business closures should be established according to the level of contact.

*“This isn’t right. What are they going to do by tracing you from 3–4 days before? They said that there’s a week of incubation period, and that some people even have 10 days of incubation. That’s why they’re isolating us like that for a month. For 15 days. I don’t think it’s right not to investigate earlier on and only investigate (contact tracing) for a few days.”*

(Participant 2)

The participants wished for individualized and practical help to alleviate the stress they had experienced during the process of COVID-19 diagnosis confirmation, isolation, and the return to their normal lives after discharge. Some participants were automatically fired for missing work, and some participants took several months to return to their job even after completely recovering from the illness.

*“As soon as I was isolated after being diagnosed, my mom basically got fired. They told her to take a leave of absence. At first, they would say they’d call her back in a month, and then that became two months, three months. She had to keep waiting.”*

(Participant 3)

Furthermore, the participants mentioned that there are negative social views about COVID-19 patients returning to their normal lives, even after they are completely recovered, and stated the need for the government to work toward changing social perceptions of COVID-19 survivors. While discussing the need for improved government disinfection policies, the participants also expressed gratitude for the government’s disinfection efforts, coverage of treatment costs, supply of basic necessities during quarantine, and consolation telephone calls.

*“Well, the public tries to avoid us. They have the wrong perception (* *about COVID-19 patients). The government should look at the data and at least say that it was okay thus far. Our country has more than 10,000 confirmed patients, right? All of them are knowingly or unknowingly restricted in their activities. They are Korean citizens, too.”*

(Participant 4)

### 3.3. Theme Cluster 3: Social Stigma: My “Scarlet Letter”

#### 3.3.1. Accidental COVID-19 Infection

The participants mentioned that they contracted COVID-19 by accident without realizing it at the time. They did not know how they contracted the virus, and after receiving a confirmed diagnosis, they tried to pinpoint the possible source of the infection in hindsight. The participants expressed that they were unfortunate to catch COVID-19, which could affect anyone, and were frustrated for not being able to avoid it. Some participants stated that they do not blame anyone because COVID-19 is no one’s fault but that they are also victims of the infection.

*“You know, it (COVID-19) is something that anyone can have in their lives.”*

(Participant 2)

#### 3.3.2. Guilt: Because of Me

The participants were constantly worried and felt guilty about their acquaintances receiving confirmed COVID-19 diagnoses and being admitted to the hospital or quarantined as well as the continued spread of the infection linked to their cases. They could not even apologize to their acquaintances.

*“If that person did not get infected by me, it might have been a little better. Maybe I would have had less of a burden. This burden was too heavy.”*

(Participant 14)

In particular, the participants were stressed by the feeling that they were the sole culprit behind the financial blow to their acquaintances’ businesses or employment, separation from their families, and inconvenience caused for important family events, such as weddings. Employed individuals felt sorry to their bosses and directors for the harm they caused to their workplace. The participants hoped not to infect anyone else and felt relieved when their acquaintances tested negative.

*“I got cursed at, and someone said that his daughter couldn’t get married on the set date because of me. A close friend of mine couldn’t even go home or stay in a hotel, and he had to stay in his car all night for several days. And a lot of my friends stayed in a hotel, and someone checked into an inn and stayed there for several days.”*

(Participant 10)

#### 3.3.3. Negatively Viewed by Others

In high-context Korean society, the participants had difficulties in interpersonal relationships due to the negative views of others. People equated them with COVID-19 itself, calling them things such as a “virus” and “virus spreader” and calling them by their confirmed case number instead of their names in public places. Some participants were annoyed by the fact that they were treated like lepers and were hurt by the COVID-19-related jokes people made inadvertently. The participants felt betrayed as their friends avoided them and talked about them behind their backs.

*“There were so many people there, and there was one person who yelled 00 number seven is here, isn’t that 00 number seven? I really wanted to hide somewhere.”*

(Participant 10)

The participants experienced the people around them being hesitant to eat, talk, or come into physical contact with them even after they were fully recovered. People were hypersensitive, avoiding riding the same elevator and raising claims unrelated to COVID-19. The negative responses from the people around them exposed the cold reality of interpersonal relationships. The participants used this as an opportunity to sort out their interpersonal network, distinguishing between people who are friendly and those who are not. Nevertheless, the participants also mentioned that they could understand the negative responses of others because COVID-19 is a first-time experience for everyone, and people do not understand it accurately.

*“I mean, they criticized me like crazy. Normally, they would say hi like this. It’s not like I’m a heretic or an anti-government terrorist or anything. This happened one time, and it’s been so hard on me. How can a person change like that all of a sudden?”*

(Participant 2)

The participants also stated that it was not necessary to disclose to other people that they were indeed the rumored COVID-19 patients. Moreover, they felt dejected as they witnessed people tense up when they disclosed that they recovered from COVID-19. In particular, the participants who proactively received a screening test stated that they would not take screening tests proactively if another outbreak occurred due to their harsh experiences with COVID-19.

#### 3.3.4. Negatively Viewed by Society

The participants became keenly aware of how society defined them as if they were sinners. Their children were rejected from private academies, their return to work was delayed as a result of irresponsible decisions made by their companies, and they were denied new health-related insurance.

The participants stated that the negative social views would likely continue until the COVID-19 pandemic is over. To them, it seems that the “scarlet letter” they have on their chest as a COVID-19 patient will never be forgotten.

*“We will only be forgotten after COVID-19 is eradicated. Before then, COVID-19 patients are just COVID-19 patients.”*

(Participant 9)

#### 3.3.5. Negative Influence of Social and Mass Media

Social and mass media rapidly spread distorted, unfiltered information about the participants and made them merely subjects of gossip. The participants suffered from a bombardment of cyber-bullying, and some even thought about committing suicide. They experienced firsthand the perils of social and mass media that escalate the severity of a situation through distorted information.

*“That is when I realized the cruelty of the press and Internet comments. From “didn’t you go to the Lotte Mall to intentionally spread the virus” to “why did you go there?” I was defined as a scumbag for intentionally spreading it. All the comments were like that in all communities, like mothers’ communities.”*

(Participant 10)

The participants were curious about the current status of COVID-19 but did not feel brave enough to look at social media or mass media and intentionally avoided them. They turned off their cellphones, avoided watching television, and even if they did watch television, they avoided the news. However, to understand the current COVID-19 situation, some participants did not miss news programs. The reason that the participants distanced themselves from social and mass media was to manage their mental health because they feared the psychological turmoil, such as anxiety and fear, it would cause them.

*“I just shut myself off intentionally and disappeared from group chats.”*

(Participant 9)

#### 3.3.6. Psychological Scars That Cannot Be Healed

Even after recovering from the illness, returning to their homes, and reentering society, the participants were still had a victim mentality because they had been COVID-19 patients and because of the emotional injury they had suffered. They wanted to erase the fact that they had been COVID-19 patients from their memory, but it persisted as a mental scar that psychologically intimidated them. They lost confidence, still feared other people’s views, and became nervous when COVID-19 was brought up as a topic. As they experienced social phobia and anxiety symptoms, such as increased heart rate and a trembling voice, they felt pity for themselves for the dramatic changes they had undergone.

*“My son tells me mom, let’s go outside, but I just hated it. I was scared and thought, wouldn’t someone recognize me? Being absorbed back into society was really difficult.”*

(Participant 1)

Further, the participants worried that they and other people would be reinfected with COVID-19. They dreaded the possibility of being quarantined and becoming the target of a social witch hunt again; thus, they always wore facemasks and practiced physical distancing.

*“I went to see my girlfriend a month later. The biggest problem was whether I could hold her hand and kiss her. I still worry that, what if I still have the virus and she gets infected?”*

(Participant 3)

*“I still armor myself when I go outside. I wear my mask and carry my sanitizer and wash my hands. Why? Because I’m scared because I had it once. You know they say people who have been beaten know it, you know I’m scared. So, I control myself even more.”*

(Participant 11)

When they felt even slightly off, the participants immediately suspected reinfection with COVID-19 or were concerned about their health. Thus, the participants strictly managed their health. For example, they used personal plates when eating, strictly followed personal hygiene practices, exercised, and took health supplements.

### 3.4. Theme Cluster 4: Mind and Body Deprived by COVID-19

#### 3.4.1. COVID-19 Symptoms

While some participants only experienced mild muscle pain for a day or two, most participants experienced typical COVID-19 symptoms, including high fever, chills, upper respiratory tract symptoms, headache, muscle pain, loss of smell and taste, hair loss, indigestion, and weakness. Some participants experienced severe symptoms, such as pneumonia and dyspnea, as well as sequelae and adverse drug reactions.

*“I didn’t know that hair loss was even a symptom. I was like, why is my hair falling out so much? It was like I was going through chemo. When I would tie my hair once, the floor would become all dark.”*

(Participant 9)

The greatest topic of interest among the participants was quickly recovering from COVID-19 without any sequelae; thus, they tried various methods they learned on their own to test negative for the virus. They tried to raise their body temperature and maintain an appropriate level of humidity in the cold and dry negative-pressure ward, and they actively cooperated with their health care providers during treatment. Some participants even employed medically unfounded strategies, such as rinsing their nose with salt and brushing their teeth with hand sanitizer, in order to receive a negative COVID-19 test.

*“They say when you go into the negative-pressure ward, keep your body warm and don’t take a shower too often. People who used to stink and not take showers were the first ones to go [be discharged], and those who washed frequently couldn’t get out.”*

(Participant 11)

#### 3.4.2. Fear of Death

As they were transported in the ambulance, the participants developed a fear of death for the first time in their lives, and when they were admitted to the hospital, they were overwhelmed by a fear of whether they would be able to walk out of the hospital alive. The anxiety triggered by COVID-19 symptoms progressed to thoughts about death. The participants felt they had come close to death when they experienced COVID-19 symptoms and adverse events from the treatment agents and were overwhelmed by the fear of death when they were alone or went to bed in a multi-patient ward. Their wards felt like the morgue, and they had nightmares about meeting the Angel of Death. They were also inundated with the feeling that they would spend the last moments of their lives in a hospital ward.

*“The moment I got in the ambulance, I thought, okay, I’m going to my deathbed. I was so sick, so I was like, okay, I’m going to die. People in the news said that you’re going to die. Oh well, let’s leave it to fate.”*

(Participant 14)

In particular, prolonged hospital stays as a result of failure to receive a negative COVID-19 test had a mental toll on the participants. Some experienced severe depression and even considered suicide. The isolated situation itself was a painful reality they wanted to avoid, and, as a result, they refused to accept telephone calls or text messages from their family or friends and lost the will to carry on. To control their suicidal ideation, the participants took tranquilizers and antidepressants and were also placed on a watchlist by their health care providers.

*“By the second month, I developed a serious depression. I wanted to jump out of the window and die, and I often had this urge to jump. I was going to die (sobbing), but I couldn’t because of my poor wife. So, I endured and endured and endured (sobbing).”*

(Participant 11)

### 3.5. Theme Cluster 5: Rediscovering Relationships through Experiencing Hardship

#### 3.5.1. Feeling Sorry for Their Family

The first thing that came to mind at the moment they were diagnosed with COVID-19 was their families; the participants could not bear the suffering their relatives had to endure just for being their family. They felt sorry for their family members as they witnessed reality abruptly stormed them, such as undergoing a screening test and being quarantined away from family once their positive diagnosis was confirmed. In particular, those who had older adults or people with underlying conditions in their family were extremely concerned and became keenly aware of this crisis.

*“My sister-in-law has cancer. That really killed me. I was caught and isolated the day before, and then, until her COVID-19 results came out, I was so psychologically pressured. I felt like dying every second, every minute. That time felt like billions of years.”*

(Participant 4)

The participants were dismayed as they watched their families suffer from a harsh reality just for being their family. In particular, the negative impact on children left a large scar on their hearts. Children who had seen their parents being taken away like a felon at the time of transport were mentally shocked and had to receive therapy. In one case, a high school senior suffered a drop in their grades due to the absence of their mother during the quarantine period. Even after returning to their normal lives, children still displayed separation anxiety, always checking to make sure their parents were there.

*“I just shoved this and that [into a bag], and I didn’t even take my scrunchies. I just shoved everything into my bag and went. I was going out, and I turned back, and my kids were just frozen (tearing up). For my kids, their mom was just gone all of a sudden, so they have this serious trauma.”*

(Participant 1)

#### 3.5.2. Family Support during Hard Times

Family support enabled the participants to push through the difficulty times experienced in isolation. The participants felt safe and supported, thanks to their families, who endured their emotional breakdowns, encouraged them, and remained beside them over the telephone when they were alone. One participant’s husband tried to calm her with white lies. The participants were moved by their family’s sacrifices and commitment as they tried hard to deliver necessities and food to them amid the strict no-visitor policy. As they experienced genuine love and care from their families, the participants were reminded of the value of family, and their relationships, including those with their spouses, were improved. However, some participants felt disappointed by family members who were inconsiderate of them, and some participants had conflicts with family members who had to take charge of all of the household chores and parenting during their spouse’s isolation.

*“My mom is 91 years old, and she would call me and ask me how I am doing. She would tell me you need to eat a lot and recover and come out. If my mom prays for me like that, then I have this hope that I can recover.”*

(Participant 14)

#### 3.5.3. Support from Others

The participants were grateful to their friends for consoling and understanding them. They were thankful that their friends and acquaintances comforted them through telephone calls and text messages during their isolation and welcomed them back when they returned to their normal lives. People helped the participants by giving them food, supplies, and money.

*“I was really thankful for that person who texted me saying not to worry because I will be fine once I get treated. Not because I received money. So, I can’t forget that in my heart, and it just makes me cry.”*

(Participant 6)

However, the participants were also extremely worried about how their colleagues would treat them once they returned to work. They felt grateful to colleagues who simply welcomed them back and comforted them as if nothing had happened. The participants also developed bonds with other patients and became supportive of each other. They felt a sense of camaraderie with their fellow patients as they asked questions about COVID-19 symptoms, shared information, and listened to and encouraged one another through social media.

*“We are in the same group and feel similar things, so we can empathize with one another. When this person says one thing, we all get mad for them and share that emotion. That’s the kind of atmosphere we have.”*

(Participant 9)

## 4. Discussion

This study aims to understand the meanings and essence of the lived experiences of COVID-19 patients in one region in South Korea, from undergoing a screening test to receiving treatment in isolation and, finally, returning to their normal lives. In this study, it was confirmed that patients infected with COVID-19 suffered more from psychosocial problems than the physical problems caused by infectious diseases. Therefore, in a high-context society like South Korea, psychosocial issues beyond simply being infected with an infectious disease such as COVID-19 can be important.

The participants expressed feeling continuous desperation amid uncertainty as they were diagnosed and treated for COVID-19, a novel disease, and underwent the recovery process with limited information. This was similar to the findings of Sun N. et al. [17], wherein the participants, who were COVID-19 patients, experienced uncertainty about the outcome of examination results during hospitalization. Desperation and uncertainty may induce psychological disturbances such as anxiety, depression, and panic disorder [18], and this study’s participants also experienced anxiety and fear of death. Jesmi et al. [19] also reported that the majority of COVID-19 patients experienced fear of death and feeling of over-dependency, in agreement with this study. Thus, infection control facilities and health care providers should alleviate patients’ uncertainty by providing valid COVID-19-related information and emotional support. Furthermore, while receiving treatment in isolation, the participants felt extreme loneliness and helplessness. Similarly, Shaban et al. [20] reported that people in quarantine due to COVID-19 had their social relationships severed, and they felt cut off from the outside world, as if time had stopped, and physically isolated and restricted. To mitigate COVID-19 patients’ loneliness and helplessness, induced by quarantine, isolation wards should be improved by installing large windows and ensuring patients have an adequate amount of space for comfort [20].

This study’s participants felt shocked and complained of being completely stripped of privacy during the disinfection process. However, studies conducted in other countries have not reported experiences related to disclosure of personal information during quarantine [8,17,19,20]. This difference can be interpreted as a difference in cultures or laws among countries. As South Korea is a high-context society, we share information necessary for the public good [12]. In addition, based on previous MERS experience, laws related to the disclosure of personal information to manage infectious diseases are being amended in South Korea [21]. While Korea’s so-called “K-Disinfection” model has been rated as a successful disinfection system, people have raised concerns about the excessive disclosure of personal information in the early days of the outbreak [3,21,22]. The participants felt embarrassed and were angered by the violation of their privacy and human rights during epidemiological investigations and the quarantine process. Therefore, with regards to personal information disclosure, it is necessary to systematically create and utilize detailed guidelines to minimize the concerns arising from it. The patients in this study had complaints about inconsistent government disinfection guidelines and confusion regarding disinfection facility procedures. This supports previous findings that patients who were subjected to at-home quarantine due to MERS were infuriated by disorganized infection control measures and developed severe mistrust toward public health authorities [23]. These results seem to be attributable to this study’s participants, having contracted the virus in the early days of the outbreak when the disinfection system was not yet firmly established. Furthermore, this suggests that the problems that have emerged throughout several epidemics in the past, such as an inadequate public health crisis response system, limited knowledge about the epidemic, and poor communication regarding disease risk [24,25], still persist. Confusion within the response system during a crisis creates widespread public mistrust toward the government, thereby hindering effective infection control. Thus, to ensure consistency in and the specificity of disinfection guidelines, it is necessary to immediately reflect on the lived experiences of patients and relevant personnel and provide adequate education and training to disinfection personnel.

The participants experienced social stigma for accidentally contracting COVID-19, in which they were viewed negatively and rejected by others in society. Social stigma from COVID-19 in South Korea’s high-context society was like a “scarlet letter” that could remain on their chests for the rest of their lives. Due to the novelty of the disease, high infection rate, and lack of effective treatments, people tend to attribute the responsibility of the outbreak to others [26,27], and the inevitable physical isolation to prevent the spread of the disease stigmatizes patients as “others”, separated from “us”, within society [28]. This study’s participants were also victims of COVID-19, and they wished to be protected and treated and, ultimately, recover from the disease; however, they could not speak up and be heard. Furthermore, even after they had physically recovered, the social stigma they suffered left a lasting scar, hindering their readjustment to society and psychologically intimidating them. Such experiences of stigma and concerns about stigmatization have an impact on the victims’ mental health, which may provoke them to make extreme choices [29], thus calling for special attention and interventions by society.

The participants mentioned the need for psychological therapy for patients and policy measures to change social perceptions. Social institutional approaches are needed to reduce social stigmatization, and, particularly, multilateral approaches are required to improve public health communication and implement promotional campaigns to improve public awareness. Furthermore, individualized psychosocial rehabilitation programs should be provided for COVID-19 patients to boost their self-esteem, confidence, and sense of belonging to improve their resilience. As social stigma has more persistent adverse psychological, emotional, and cognitive effects on individuals than physical injuries [30], qualitative studies should be conducted to gain an in-depth understanding of the stigma experienced by COVID-19 patients.

In contrast to past outbreaks, COVID-19 induced an infodemic (i.e., an overabundance of incorrect information and fake news), which escalated public anxiety and caused the public to put their faith in ineffective treatments and to refuse to comply with preventive measures while stirring up prejudice [31] and causing social anomie. The findings of this study also support the existence of a problem with distorted information being spread through social media and the press. Based on such distorted information, the participants were suspicious of screening test results, displayed frustration regarding their separation from health care providers and strict restrictions, and had inaccurate perceptions about asymptomatic conditions and isolation. Moreover, the participants engaged in medically unfounded health behaviors to facilitate their conversion to a negative result and quick recovery. During the COVID-19 pandemic, health care providers play a crucial role and should provide infection control education to patients and survivors based on close observation and counseling in order to correct any erroneous knowledge and perception. To this end, health care providers should have good communication skills during a crisis.

COVID-19 patients participating in this study felt sorry to their family members who suffered as a result of their infection, and they were grateful that their family still remained steadfast supporters regardless of such hardships. Infections such as COVID-19 impact all members of a family [17], so it is necessary to develop and implement family-targeted empowerment programs. In addition, the participants felt warmth and gratitude toward people who comforted and understood them while they were hurt by those who rejected and avoided them; on this basis, they re-established their interpersonal network. This indicates that positive support from others is an important social resource that promotes the recovery of COVID-19 patients in order to return to their everyday lives.

Thus, this study is significant in that it provides in-depth data for understanding the experiences of patients who contracted COVID-19 in the early days of the pandemic, increasing awareness of the need to provide psychosocial rehabilitation to improve patient resilience and develop relevant interventions.

## 5. Conclusions

This study presents an in-depth understanding of and describes the physical, psychological, and social problems experienced by COVID-19 patients based on their lived experiences. In particular, social stigma is a barrier to the patients’ return to their normal lives in South Korea’s high-context society. Therefore, individualized and comprehensive psychosocial rehabilitation programs should be implemented to improve these patients’ resilience. Subsequently, qualitative studies on the stigma experienced by COVID-19 patients and longitudinal studies on their resilience are needed.

## Figures and Tables

**Table 1:** Demographic and COVID-19-related data of participants.

Participant	Age/Gender	Marital Status	Education	Religion	Employed	Underlying Medical Condition	Smoking	Route of Infection	Hospitalization Period	Clinical Symptoms at the Time of Admission	After Effects
1	45/F	Married	University	None	Yes	Hyperlipidemia	Never	Co-worker	19 days	Cough, sputum, sore throat	Fatigue, hair loss, aches in the back of the head
2	64/M	Married	University	Yes	Yes	None	None	Overseas entry	25 days	Body aches, loss of appetite, loss of smell	None
3	25/M	Single	High school	None	None	None	None	Religious meeting	24 days	Headache, mild fever	Intermittent headache
4	56/M	Married	High school	None	Yes	None	None	Religious meeting	30 days	Asymptomatic	Hair loss, fatigue, palpitations, fear of covid-19 reinfection
5	42/M	Married	University	Yes	Yes	None	None	Overseas entry	36 days	Sore throat, dry cough	Memory loss, chest tightness, headache
6	70/M	Married	Elementary school	Yes	None	Hypertension, prostatic hyperplasia	None	Religious facilities	20 days	Asymptomatic	Shortness of breath while doing some activities
7	22/M	Single	University	Yes	None	None	Smoking	Visited COVID-19-prevalent area	50 days	Muscle pain	Shortness of breath
8	73/M	Married	High school	None	None	Hypertension, diabetes mellitus	None	Accompanied family member	24 days	Muscle pain, cough, runny nose	Joint pain, fatigue
9	48/F	Married	University	None	Yes	Hypertension, retinal obstruction	None	Accompanied family member	40 days	Mild fever, sore throat	Fatigue, hair loss, sleep disorder
10	49/M	Married	University	None	Yes	Reflux esophagitis, allergic rhinitis	None	Unknown	7 days	Body ache, muscle pain	None
11	68/M	Married	High school	Yes	None	Hypertension	None	Accompanied family member	54 days	Headache	Fatigue, body ache after exercise, muscle pain, loss of smell, smell of burning food
12	40/F	Married	University	None	None	None	None	Contact with a COVID-19 confirmed patient	53 days	Muscle pain, sputum	Fatigue, poor concentration, loss of smell and taste; fishy smell, sesame oil smell
13	70/F	Married	Elementary school	Yes	None	Hypertension, hyperlipidemia	None	Religious meeting	17 days	Sore throat	Fatigue
14	67/F	Widowed	Middle school	Yes	Yes	Hypertension	None	Religious meeting	33 days	Cough, fever	Fatigue, hair loss, shortness of breath
15	66/F	Married	Middle school	Yes	Yes	Heart failure, diabetes mellitus	None	Religious meeting	90 days	Cough, body ache	Joint pain, fatigue, hair loss
16	67/F	Married	Middle school	Yes	Yes	None	None	Unknown	24 days	Body ache	None

**Table 2:** COVID-19 patients’ physical and psychological experiences.

Theme Clusters	Theme	Significant Statement
Desperate and uncertain times during COVID-19 diagnosis and treatment	Feeling Anxious about COVID-19 diagnosis	- Suspected COVID-19 infection - Proactive preemptive screening - Hoping not to be confirmed as COVID-19 positive - Unpredictability of COVID-19 infection - Feelings around being a confirmed COVID-19 patient - Confirmed COVID-19 positive
	Terrifying life in quarantine room with everything blocked	- Confined to a hospital room - Feeling of loneliness and sadness - Completely disconnected from the outside - A meaningful time to reflect on life - The season changed when I was relieved from isolation
	Conquered by helplessness	- Living in a hospital room without doing anything is boring - Repeated daily routine life - There’s nothing you can do by yourself - Exhausted from the long-term hospitalization - Trying to find something that you can do
	Appreciation for medical personnel	- Feeling grateful to the healthcare provider - Relying on the kind and helpful medical personnel - Want to be helpful to nurses in need
Shock and complaints related to the disinfection process	Being completely stripped of privacy during the disinfection process	- Suddenly stuck in the hospital/sudden isolation in hospital - Private life is completely revealed - A rumor that I was confirmed COVID-19 spread through the whole town - Epidemiological investigation is compelling - COVID-19 confirmed patients must be protected from privacy infringement
	Unorganized disinfection system	- Even public officials are unaware of proper procedures - Practitioners use the quarantine guidelines without context - Sustained damages due to errors in the quarantine system - Repeated epidemiological investigation from the relevant institutions
	Need for government policies	- It is necessary to prepare quarantine standards according to the situation - Psychological treatment that is practically helpful is needed - Support for livelihoods of the confirmed patients is required - COVID-19 awareness needs to be improved
Social stigma: My scarlet letter	Accidental COVID-19 Infection	- Unfortunately, I was infected with COVID-19 - Exposed to COVID-19 without knowing it - It would have been nice if we had avoided the infectious circumstances - As a COVID-19 patient, I am a victim too
	Guilt: Because of me	- Feeling guilty for causing more Covid-19 infections - Causing harm to my acquaintances - Feeling sorry for infecting co-workers with COVID-19 - Feeling relieved by the COVID-19 negative test results of others
	Negatively viewed by others	- I was treated like a virus - I was called by my COVID-19 confirmation number instead of my name - Negative response and ignorance from others - Backtalk and criticism from others - Feeling betrayed by others - There is no need to be intentionally notified of confirmed positive result
	Negatively viewed by society	- COVID-19 confirmed patients exposed to prejudice - My children were rejected from their academy - Irresponsible workplace attitude was imposed on me
	Negative influence of social and mass media	- Social media spreads exaggerated and distorted information - Suffering from inappropriate comments on social media - Afraid of following social and mass media - Intentionally avoiding social and mass media
	Psychological scars that cannot be healed	- Psychological atrophy and anxiety persist even after full recovery from COVID-19 - Worrying about reinfection - Keeping to quarantine regulations preemptively - Potential anxiety about health
Mind and body deprived by COVID-19	COVID-19 symptoms	- Asymptomatic - Various symptoms and side effects - Drug side effects - Efforts to heal the disease
	Fear of death	- Feeling that death is near - An opportunity to think about the meaning of life and death - Late conversion from positive COVID-19 result to negative result - Psychological anxiety and depression
Rediscovering relationships through experiencing hardship	Feeling sorry for their family	- Concerned about family infection - Feeling of crisis due to family infection - Heartache following harm caused to family
	Family support during hard times	- I was able to withstand the difficulties, thanks to the family - Feeling thankful for the care and support from the family - Improving family relationships
	Support from others	- Consolation and help of friends and acquaintances - Feeling thankful for neighbors - Comfort and understanding of colleagues at work - Forming consensus with COVID-19-confirmed colleagues

## Data Availability

The data presented in this study are available on request from the corresponding author. The data are not publicly available due to ethical and privacy reasons.

## References

[1] Heo J.Y. (2020). Clinical and Epidemiological Characteristics of Coronavirus Disease 2019 in the Early Stage of Outbreak. Korean J. Med..

[2] Zhu N., Zhang D., Wang W., Li X., Yang B., Song J., Zhao X., Huang B., Shi W., Lu R. (2020). A Novel Coronavirus from Patients with Pneumonia in China, 2019. N. Engl. J. Med..

[3] Kim J.E., Lee J.H., Lee H., Moon S.J., Nam E.W. (2021). COVID-19 screening center models in South Korea. J. Public Health Policy.

[4] Song Y., Zhang M., Yin L., Wang K., Zhou Y., Zhou M., Lu Y. (2020). COVID-19 treatment: Close to a cure? A rapid review of pharmacotherapies for the novel coronavirus (SARS-CoV-2). Int. J. Antimicrob. Agents.

[5] Lee S.Y. (2020). Policy Directions and Challenges for COVID-19 Quarantine Response. Health Welf. Issue Focus.

[6] Xiang Y.T., Yang Y., Li W., Zhang L., Zhang Q., Cheung T., Ng C.H. (2020). Timely Mental Health Care for the 2019 Novel Coronavirus Outbreak Is Urgently Needed. Lancet Psychiatry.

[7] Bo H.-X., Li W., Yang Y., Wang Y., Zhang Q., Cheung T., Xiang Y.-T. (2020). Posttraumatic Stress Symptoms and Attitude Toward Crisis Mental Health Services among Clinically Stable Patients with COVID-19 in China. Psychol. Med..

[8] Sahoo S., Mehra A., Suri V., Malhotra P., Yaddanapudi L.N., Dutt Puri G., Grover S. (2020). Lived Experiences of the Corona Survivors (Patients Admitted in COVID Wards): A Narrative Real-Life Documented Summaries of Internalized Guilt, Shame, Stigma, Anger. Asian J. Psychiatry.

[9] Jamieson A. Why Some People Feel Guilty After Testing Positive for COVID-19. https://www.healthline.com/health-news/why-some-people-feel-guilty-after-testing-positive-for-covid-19.

[10] Bendau A., Petzold M.B., Pyrkosch L., Mascarell Maricic L., Betzler F., Rogoll J., Plag J. (2020). Associations Between COVID-19 Related Media Consumption and Symptoms of Anxiety, Depression and COVID-19 Related Fear in the General Population in Germany. Eur. Arch. Psychiatry Clin. Neurosci..

[11] Choi S.-H. (2020). Preventive Measures during Outbreak of Coronavirus Disease 2019. Korean J. Med..

[12] Thomas J. (1998). Contexting Koreans: Does the High/Low Model Work?. Business Commun. Quart..

[13] Neubauer B.E., Witkop C.T., Varpio L. (2019). How Phenomenology Can Help Us Learn from the Experiences of Others. Perspectives Med. Educ..

[14] Colaizzi P., Valle R.S., King M. (1978). Psychological Research as a Phenomenologist Views It. Existential Phenomenological Alternatives for Psychology.

[15] Sandelowski M. (1986). The Problem of Rigor in qualitative research. Adv. Nurs. Sci..

[16] Tong A., Sainsbury P., Craig J. (2007). Consolidated Criteria for Reporting Qualitative Research (COREQ): A 32-Item Checklist for Interviews and Focus Groups. Int. J. Quality Health Care.

[17] Sun N., Wei L., Wang H., Wang X., Gao M., Hu X., Shi S. (2021). Qualitative Study of the Psychological Experience of COVID-19 Patients During Hospitalization. J. Affect. Disord..

[18] Fofana N.K., Latif F., Sarfraz S., Bashir M.F., Komal B. (2020). Fear and Agony of the Pandemic Leading to Stress and Mental Illness: An Emerging Crisis in the Novel Coronavirus (COVID-19) Outbreak. Psychiatry Res..

[19] Jesmi A.A., Mohammadzade-Tabrizi Z., Rad M., Hosseinzadeh-Younesi E., Pourhabib A. (2021). Lived Experiences of Patients with COVID-19 Infection: A Phenomenology Study. Med. Glas..

[20] Shaban R.Z., Nahidi S., Sotomayor-Castillo C., Li C., Gilroy N., O’Sullivan M.V.N., Sorrell T.C., White E., Hackett K., Bag S. (2020). SARS-CoV-2 Infection and COVID-19: The Lived Experience and Perceptions of Patients in Isolation and Care in an Australian Healthcare Setting. Am. J. Infect. Control.

[21] Kim H.E. (2020). Coronavirus Privacy: Are South Korea’s Alerts Too Revealing. BBC News Korean.

[22] Lee J. (2020). COVID-19 and Korea: Emerging Cultural “Borders” and Biopolitics in Times of Extremity. J. Inst. Social Sci..

[23] Ha J.-Y., Ban S. (2017). Home Isolation Experience of the People Exposed to Middle East Respiratory Syndrome Positive Patients. J. Qual. Res..

[24] Cha K.S., Shin M.J., Lee J.Y., Chun H.K. (2017). The Role of Infection Control Nurse during Emerging Infectious Disease Epidemic: Focusing on the Middle East Respiratory Syndrome. Korean J. Healthc. Assoc. Infect. Control Prev..

[25] Sarikaya O., Erbaydar T. (2007). Avian Influenza Outbreak in Turkey through Health personnel’s Views: A Qualitative Study. BMC Public Health.

[26] Bhattacharya P., Banerjee D., Rao T.S. (2020). The “Untold” Side of COVID-19: Social Stigma and its Consequences in India. Indian J. Psychol. Med..

[27] Shreyaswi Sathyanath M., Shashwath Sathyanath M. (2020). Stigma Reduction and Provision of Mental Health Services in the Public Health Response to COVID-19. Indian J. Community Health.

[28] Rubin G.J., Wessely S. (2020). The Psychological Effects of Quarantining a City. BMJ.

[29] Bhattacharya P., Banerjee D. (2020). “The Fear Within...”: Dimensions of Social Stigma, Othering and Orientalism during COVID-19 in India. Int. Soc. Secur. Rev..

[30] Almutairi A.F., Adlan A.A., Hanan H., Balkhy H.H., Oraynab A., Abbas O.A., Clark A.M. (2018). “It Feels Like I’m the Dirtiest Person in the World.”: Exploring the Experiences of Healthcare Providers who Survived MERS-CoV in Saudi Arabia. J. Infect. Public Health.

[31] Banerjee D., Sathyanarayana Rao T.S. (2020). Psychology of Misinformation and the Media: Insights from the COVID-19 Pandemic. Indian J. Soc. Psychiatry.

